# HM30181A, a potent P-glycoprotein inhibitor, potentiates the absorption and *in vivo* antitumor efficacy of paclitaxel in an orthotopic brain tumor model

**DOI:** 10.20892/j.issn.2095-3941.2020.0128

**Published:** 2020-12-15

**Authors:** Wu Zeng, Betty Yuen Kwan Law, Vincent Kam Wai Wong, Denise So Bik Chan, Simon Wing Fai Mok, Joyce Jia Ying Gao, Rebecca Ka Yan Ho, Xu Liang, Jia Hao Li, Ming Tsung Lee, Weng Li Yoon, Michael P Smolinski, Johnson Yiu Nam Lau, Christopher Wai Kei Lam, Manson Fok

**Affiliations:** 1 State Key Laboratory of Quality Research in Chinese Medicines, Macau University of Science and Technology, Macau, China; 2 Athenex Hong Kong Innovative Limited, Hong Kong, China; 3 Faculty of Medicine, Macau University of Science and Technology, Macau, China; 4 Athenex Inc., New York 14203, USA

**Keywords:** HM30181A, glioma, pharmacokinetics, paclitaxel, P-glycoprotein

## Abstract

**Objective:**

Delivery of chemotherapeutic drugs to the brain has remained a major obstacle in the treatment of glioma, owing to the presence of the blood-brain barrier and the activity of P-gp, which pumps its substrate back into the systemic circulation. The aim of the present study was to develop an intravenous formulation of HM30181A (HM) to inhibit P-gp in the brain to effectively deliver paclitaxel (PTX) for the treatment of malignant glioma.

**Methods:**

Two formulations of solubilized HM were designed on the basis of different solid dispersion strategies: i) spray-drying [polyvinlypyrrolidone (PVP)-HM] and ii) solvent evaporation [HP-β-cyclodextrin (cyclodextrin)-HM]. The P-gp inhibition of these 2 formulations was assessed on the basis of rhodamine 123 uptake in cancer cells. Blood and brain pharmacokinetic parameters were also determined, and the antitumor effect of cyclodextrin-HM with PTX was evaluated in an orthotopic glioma xenograft mouse model.

**Results:**

Although both PVP-HM and cyclodextrin-HM formulations showed promising P-gp inhibition activity *in vitro*, cyclodextrin-HM had a higher maximum tolerated dose in mice than did PVP-HM. Pharmacokinetic study of cyclodextrin-HM revealed a plasma concentration plateau at 20 mg/kg, and the mice began to lose weight at doses above this level. Cyclodextrin-HM (10 mg/kg) administered with PTX at 10 mg/kg showed optimal antitumor activity in a mouse model, according to both tumor volume measurement and survival time (*P* < 0.05).

**Conclusions:**

In a mouse orthotopic brain tumor model, the intravenous co-administration of cyclodextrin-HM with PTX showed potent antitumor effects and therefore may have potential for glioma therapy in humans.

## Introduction

Malignant glioma or glioblastoma multiforme composes the subset of primary brain tumors with the highest mortality rate and is associated with very poor prognosis. In the USA, the current 5- and 10-year survival rates are 4.5% and 2.7%, respectively^1^, thus underscoring the requirement for more effective therapeutic strategies for patients with glioblastoma. Paclitaxel (PTX) is a highly potent antitumor drug for the management of various types of cancer, including glioblastoma multiforme and brain metastases. However, the multidrug resistant P-glycoprotein (P-gp) is highly expressed in the capillary endothelial cells and microvasculature in the brain. Because PTX is a P-gp substrate, PTX in the brain can be removed quickly by P-gp and re-enter the systemic circulation; this pathway is hypothesized to be an important factor limiting the activity of PTX in patients with glioblastoma multiforme and brain metastases^2^. Moreover, because the oral availability of PTX is low^3^, many studies over the past few decades have focused on improving the bioavailability of PTX. The intestinal uptake of PTX, beyond being influenced by poor aqueous solubility and oral availability, is impeded *via* the active efflux transporter P-gp^4^, thus limiting its usage in targeting neuroblastoma^5^. The combined use of P-gp inhibitors with PTX may therefore improve the pharmacokinetic (PK) profile of PTX by increasing the drug concentrations in the brain tissue and thus in brain tumors.

A recent study has reported that co-administration with the P-gp inhibitor HM30181A (HM) increases the oral bioavailability and efficacy of PTX^6^. HM, compared with the similar P-gp inhibitor XR9576 (Tariquidar)^7^, is ~24 times more potent in inhibiting P-gp-mediated transport of rhodamine 123 (Rho123) from human 293FRT embryonic kidney cells stably expressing ATP binding cassette subfamily B member 1 (ABCB1)^8^. Additionally, oral administration of HM (10 mg/kg) has been found to increase the bioavailability of PTX from 3% to 41% in rats^8^. HM has been hypothesized to be a promising compound for the development of an intravenous (i.v.) formulation to inhibit P-gp activity and allow drugs to cross the blood-brain barrier. Because HM is a relatively insoluble compound, the formulation of HM for i.v. administration is challenging. The aims of the present study were two-fold: first to develop an i.v. formulation of HM to inhibit the P-gp transporter and allow drugs to cross into the brain; second to determine whether this approach with HM might enhance the penetration of PTX into the brain tumor tissue and to assess its potential in the treatment of glioblastoma multiforme. We successfully developed 2 formulations of i.v. HM by using spray-drying and solvent evaporation approaches with polyvinylpyrrolidone (PVP) and cyclodextrins, respectively, as the key functional excipients. PVP is a water-soluble polymer that has been used as a ‘drug polymer carrier’ in solid dispersion systems^9^, whereas cyclodextrins are characterized by their high bio-adaptability, which alleviates the unfavorable insolubility of drug molecules through the formation of inclusion complexes or cyclodextrin-drug conjugates^10^.

## Materials and methods

### Spray-drying preparation of the PVP-HM formulation

HM was purchased from Hanmi Fine Chemical Co. Ltd. The HM spray solution was prepared by first dissolving HM through mixing and sonication in an aqueous-organic solvent comprising dichloromethane (ACS, Wilmington, DE, USA), ethanol (ACS, Wilmington, DE, USA), and water. The polymer carrier PVP (Kollidon^®^ 12; BASF Corporation) was subsequently added under continuous mixing and sonication until the HM was completely dissolved. The spray solution was added to a Buchi Spray Dryer B-290 (BÜCHI Labortechnik AG, Flawil, Switzerland) and atomized through the nozzle with the following spray conditions: inlet temperature of 100 °C, outlet temperature of 61 °C, and flow rate of 15%, with aspiration of 100%. The resultant PVP-HM powdered solid dispersion was subsequently stored in an airtight container. Before use, the powdered dispersion was dissolved in purified water containing 5% dextrose.

### Solvent evaporation preparation of HP-β-cyclodextrin (cyclodextrin)-HM

Through the solvent evaporation approach, HM was dissolved in warmed ethanol (ACS, Wilmington, DE, USA), and cyclodextrin (DELI Biological Chemical, Xi’an, China) was then added with gentle mixing. The mixture was incubated in a water bath (65 °C) to allow the residual ethanol to evaporate, thus leading to the formation of a solid dispersion. The solid dispersion was reconstituted with 20 mL distilled water and vortexed to form a clear solution. Finally, the solution was filtered through a 0.22-μM nylon filter and stored at 4 °C.

### Design of the i.v. HM formulation

Two strategies (i.e., spray drying and solvent evaporation) were used for preparing the HM solid dispersion. For the spray-drying process, Kollidon^®^ 12, a PVP, was selected as the ‘polymer carrier,’ owing to its lower molecular weight and hence lesser propensity for kidney retention, as well as its solubility in both aqueous and organic solvent systems, which aided in the preparation process for the spray solution containing HM. Furthermore, during the vapor condensation phase in the spray-drying process, the polymer tended to form an ‘amorphous’ state with HM as a drug-polymer dispersion, hence further increasing its aqueous solubility. The HM spray-dried powder was dissolved in purified water containing 5% dextrose before use. With the solvent evaporation approach, cyclodextrin played a critical role in preparation of the HM i.v. injection. Because cyclodextrin has a bucket-like structure with an internal hydrophobic cavity and an external hydrophilic surface, the preparation process involved mixing cyclodextrin with HM in ethanol to facilitate the formation of an inclusion complex by accommodating HM within its cavity^11^. Consequently, after evaporation of ethanol, the recrystallization of HM was inhibited. After completion of the evaporation process, HM was dispersed molecularly in solid cyclodextrin, thus forming a powdered formulation ready for reconstitution with distilled water. Stability studies of reconstituted solutions for both formulations also confirmed that the injections were stable for at least 1 week when stored at 4 °C, and no precipitation of HM was observed.

### Preparation of the PTX i.v. solution

PTX was purchased from Chongqing Taihao Pharmaceutical Co. Ltd.. The i.v. PTX dosing solution was freshly prepared before use by dilution of PTX in a vehicle solution comprising 13.3% ethanol and 13.3% Cremophor EL (Sigma-Aldrich, St. Louis, MO, USA) in 5% dextrose. Controls for the corresponding drug were prepared through the same procedure described above, by using vehicle solution without PTX.

### Cell culture

U-87 MG-luc2 human glioblastoma tumor cells of unknown origin (Caliper; PerkinElmer, Inc.), authenticated by STR profiling, were maintained *in vitro* in monolayer culture in EMEM (Thermo Fisher Scientific, Waltham, MA, USA) supplemented with 10% heat inactivated FBS, 100 U/mL penicillin, and 100 μg/mL streptomycin at 37 °C in a humidified incubator with 5% CO_2_. Cells in exponential growth phase were harvested and counted for tumor inoculation.

### Rho123 accumulation assay

P-gp activity was determined by measuring the intracellular accumulation of Rho123 in A549 taxol-resistant human lung cancer cells (KeyGen Biotech, Nanjing, China), and a well-known P-gp inhibitor, 10 μM verapamil (Sigma-Aldrich, St. Louis, MO, USA), was used as the positive control. The U-87 MG cell lines were plated in 6-well plates and transfected with 2 μg ABCB1 plasmid with Lipofectamine^®^ LTX kit (Thermo Fisher Scientific, Waltham, MA, USA) for 8 h. Cells were pre-treated with the 2 HM formulations for 4 h at 37 °C before incubation with 5 μg/mL Rho123 at 37 °C for 1 h. The Rho123 accumulation assay was terminated by washing the cells with ice-cold PBS (Invitrogen, Carlsbad, CA, USA) and re-suspending them in 100 μL PBS. The intracellular Rho123 signal was detected with flow cytometry (FACSAria™ III flow cytometer; BD Biosciences, CA, USA) at an excitation wavelength of 488 nm and an emission wavelength of 525 nm. Data were analyzed in FlowJo v10.6.1 software (BD Biosciences, CA, USA) and are expressed as a percentage of Rho123 positive cells.

### Toxicity studies

Animals used for all experiments were C57BL/6 mice, 6–8 weeks of age, which were purchased from The Chinese University of Hong Kong (Hong Kong, China). The mice were administered with either cyclodextrin-HM or PVP-HM *via* i.v. injection once per week at 5, 10, 15, 20, 25, 30, and 35 mg/kg (*n* = 3 for each dosage) for the determination of the maximum tolerated doses of HM formulations. The body weights and physical conditions were recorded every day for 14 days. The experiments were performed in accordance to the “Institutional Animal Care and User Committee guidelines” of Macau University of Science and Technology.

### Bio-layer interferometry analysis

Albumin was biotinylated with EZ-Link NHS-LC-LC-Biotin (Thermo Fisher Scientific, Waltham, MA, USA) for 30 min at room temperature and then added to a 96-well plate (Greiner Bio-One, PN:655209). Biotinylation was performed by loading the reaction complex onto a super streptavidin capacity probe (ForteìBIO, Menlo Park, CA, United States), and results were analyzed with a FortéBIO Octet Red instrument upon binding dPTX or HM. ForteìBIO data analysis software was used to calculate the association and dissociation kinetic constants.

### PK analyses of cyclodextrin-HM

C57BL/6 mice were divided into 3 groups (*n* = 3) receiving different doses of cyclodextrin-HM (10, 15, or 20 mg/kg). Plasma samples were obtained from mice at 0, 0.083, 0.25, 0.5, 1, 2, 4, 8, and 24 h time points. Non-compartmental analysis of plasma data after intravenous bolus input was used to determine standard PK parameters of analytes. Administration i.v. has the advantage of allowing for rapid exposure as well as precise control of dose and administration of the tested therapeutic compounds^12^, and oral administration may be less efficient with low drug bioavailability, owing to drug adsorption and metabolization in the liver. Therefore, we used i.v. through the tail vein to increase the bioavailability of cyclodextrin-HM and to reduce the volume of administration, thus facilitating rapid absorption, distribution, and exposure to different organs. Mice were then anesthetized through intraperitoneal injection of pentobarbital (50 mg/kg) before collection of blood for further analysis.

### Co-treatment with cyclodextrin-HM and PTX by using dosing intervals

The optimal dosing interval of PTX post-HM administration was determined in C57BL/6 mice. The animals were first injected i.v. with HM (15 mg/kg) *via* the tail vein, and PTX (10 mg/kg) was administered at 0.25, 0.5, 0.75, 1, 2, 4, or 8 h post-HM administration (*n* = 3 per time point). The plasma and brain samples were collected 0.5 h after PTX was administered. The brain/blood ratio has emerged as a preferred method for evaluating the brain-targeting efficiency of neurotherapeutics with the CNS PKs in terms of brain bioavailability^13^. Therefore, the concentration of PTX in the brain divided by the concentration of PTX in the blood at different sample collection time points was calculated as described in the literature^14^.

### Co-treatment with cyclodextrin-HM and PTX for brain penetration

C57BL/6 mice were subjected to a time course study with co-treatment with HM and PTX *via* tail vein injection. Mice were first administered with HM (15 mg/kg), whereas an equal volume of vehicle formulation without HM was administered to the control group. PTX was injected into the mice 30 min post-HM administration at each time point, at 10 mg/kg. Plasma and brain samples were collected at 0.5, 1, 2, 4, 6, 8, 24, 48, and 72 h after the co-treatments.

### Orthotopic brain tumor model

An orthotopic brain tumor model was established by Wuxi Apptec. Briefly, female BALB/c nude mice 6–8 weeks of age and weighing 18–22 g (Shanghai Sippr-BK Laboratory Animal Co. Ltd., Shanghai, China) were anesthetized through intraperitoneal injection of pentobarbital sodium (80 mg/kg) and subcutaneously administered 0.1 mg/kg buprenorphine before and after surgery for analgesia. The anesthetized mice were intracranially (i.c.) inoculated with U-87 MG-Luc2 cells (2×10^5^ cells in 2 μL PBS) in the right frontal lobe. Treatments started 6 days after tumor inoculation by tail vein injection of HM and PTX (q4d×5). The physiological condition of the animals was assessed daily. The animals were pre-anesthetized with a mixture gas of oxygen and isoflurane (1%–3%) to achieve a complete anesthetic state before bioluminescence measurements. Tumor growth was monitored with an *in vivo* imaging system (Lumina III; PerkinElmer, Inc.), and the survival of all animals was recorded for the calculation of the median survival time (MST) for each group. The increase in lifespan (ILS) was determined as the ratio of the MST of the treatment group to that of the control group, expressed as the percentage increase over the lifespan of the control animals.

### Liquid chromatography-mass spectrometry (LC-MS) analysis

Brain and plasma samples collected from mice were spiked with glibenclamide (5 μg/mL) as an internal standard and subjected to serial extraction with methanol and collection of the supernatant. The brain samples were homogenized through ultrasonication in saline before the extraction procedure. The collected supernatant was dried under nitrogen gas, reconstituted in acetonitrile, and divided into aliquots that were loaded onto the LC-MS/MS system. The compounds in the plasma and brain samples were fractionated with an Agilent Zorbax Eclipse Plus C-18 column (Agilent Technologies, Inc.) with a particle size of 1.8 μm (flow rate, 0.35 mL/min). The mobile phase for measurement of all compounds was phase A (0.1% formic acid in water) and phase B (0.1% formic acid in acetonitrile): 0–3 min/2%–10% B; 3–8 min/10%–60% B; 8–13 min/60%–90% B; 13–14 min/90%–2% B; and 14–15 min/2% B. The column and auto-sampler temperatures were maintained at 40 °C and 4 °C, respectively. Data were analyzed in Agilent MassHunter workstation software vB.01.03 (Agilent Technologies, Inc.). The gas temperature was set at 325 °C (flow rate of 10 L/min), and the pressure was 40 psi for the nebulizer with a capillary voltage of 4,000 V. The fragmentor voltages were 140, 105, and 120 V for HM, PTX, and glibenclamide, respectively. The mass transitions were set as follows in multiple reaction monitoring mode: m/z 689.2 to 541.2 for HM; 854.2 to 286.1 for PTX; and 494.1 to 369.0 for glibenclamide. Quantification was performed through selected reaction monitoring of the product ions of HM and PTX. The amounts of the 2 drugs in the plasma and brain samples collected from mice were quantified by using standard and linear least-squares regression curves through calculation of the area under curve (AUC). Validation of the method is shown in **Supplementary Table S1**.

### PK analysis

PK analysis was performed with PKSolver (Microsoft Corporation)^15^. The procedure used a freely available menu-driven add-in program for Microsoft Excel written in Visual Basic for Applications. PK analysis was performed through non-compartmental analysis according to Bailer’s method^16^.

### Statistical analysis

Statistical analyses were performed in Microsoft Excel (Microsoft Corporation) and Prism (GraphPad Software). The statistical significance for all experiments was determined with one way ANOVA and Dunnett test. Kaplan-Meier statistical analysis was used for the calculation of the survival rates of different groups of mice, each containing 8 animals, and P-values were calculated with the log rank test. Differences were considered statistically significant when the *P*-value was less than 0.05.

## Results

### P-gp inhibition potential of i.v. HM formulations

**Figure 1:**
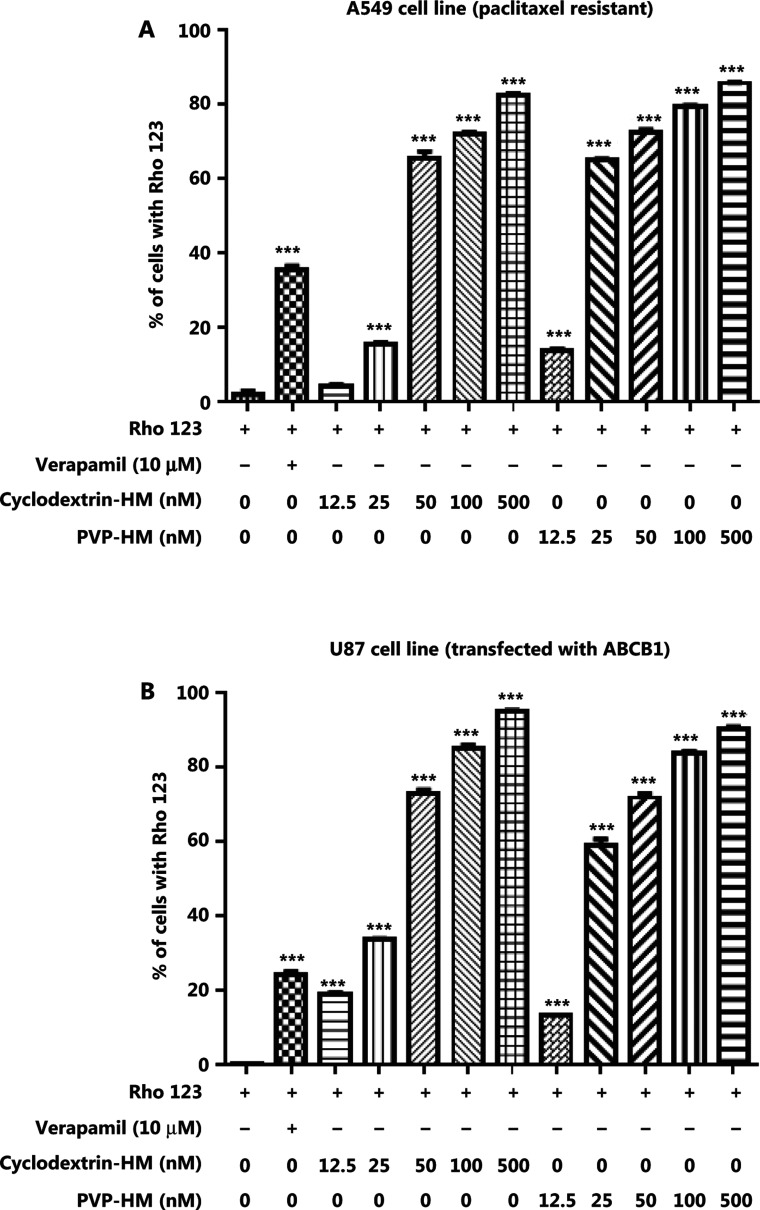
Inhibition of the expression of P-glycoprotein on the cell membranes of A549 cells and ABCB1 overexpressing U-87 MG cells by HM. PTX-resistant A549 cancer cells or ATP binding cassette subfamily B member 1 overexpressing U-87 MG cells were incubated with DMSO (negative control), verapamil (10 μM), and HM (diluted in HP-β-cyclodextrin or polyvinylpyrrolidone) at the indicated concentrations for 4 h. (A) Bar chart quantifying the accumulation of Rho123 dye in the PTX-resistant A549 cancer cells in Rho123 exclusion assays. All data are presented as mean ± SEM; *n* = 3. *P < 0.05, **P < 0.01, ***P < 0.001 (one-way ANOVA). (B) Mean fluorescence intensity of Rho123 in ABCB1 overexpressing U-87 MG cells detected by flow cytometry.

Increased expression of the ABCB1 gene, which encodes the membrane drug transporter P-gp, is a well-known mechanism for the development of drug resistance to several frontline chemotherapeutics, such as doxorubicin and PTX^17^. To evaluate the P-gp inhibition potential of HM, we incubated A549 taxol-resistant human lung cancer cells and ABCB1 overexpressing U-87 MG cell lines with increasing concentrations of HM (12.5, 25, 50, 100, and 500 nM) prepared with either PVP-HM or cyclodextrin-HM; verapamil (10 μM) served as the positive control. Rho123 fluorescent dye was used as the P-gp substrate probe for measuring the efficacy of HM in inhibiting P-gp, as reflected by the percentage of cells with accumulation of Rho123 in P-gp-overexpressing cells. This assay therefore allowed for the measurement of P-gp inhibitory potential on the basis of the P-gp-mediated transport of Rho123 dye^18^. As shown in **Figure 1A** and **B**, 10 μM verapamil increased the percentage of Rho123 positive cells from 0.99% to 36.5% in A549 cells (**Supplementary Figure S1A**), and 0.44% to 26% in U-87 MG cells (**Supplementary Figure S1B**), thus indicating successful inhibition of P-gp activity. Addition of HM, even at a low concentration (12.5 nM), promoted the uptake of Rho123 by A549 cells and the ABCB1 overexpressing U-87 MG cell line, thus increasing Rho123 positive cells in a dose-dependent manner. These results suggested that HM formulated as PVP-HM and cyclodextrin-HM has high potency in inhibiting P-gp activity.

### Maximum tolerated doses of HM formulations

**Figure 2:**
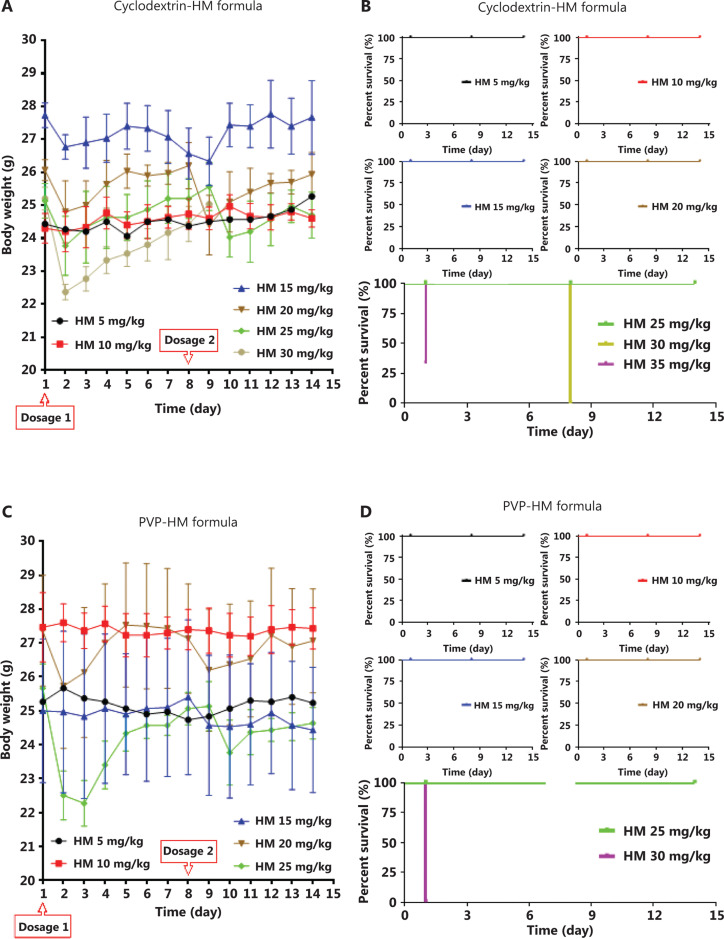
Toxicity of different dosages of cyclodextrin-HM and PVP-HM formulas after i.v. administration. Mice were administered different HM formulae *via* i.v. injection once per week (days 1 and 8) at doses of 5, 10, 15, 20, 25, 30, and 35 mg/kg. The body weight and physical conditions were recorded for 14 days. (A and B) Body weight and survival times of mice after treatment with the cyclodextrin-HM formula. (C and D) Body weight and survival time of mice after treatment with the PVP-HM formula. All data are presented as mean ± SEM; *n* = 3.

To assess the tolerance to the 2 formulations *in vivo*, we administered 2 doses of PVP-HM or cyclodextrin-HM injection on days 1 and 8, respectively, with doses ranging from 5 to 35 mg/kg, to each treatment group of C57BL/6 mice. The body weights of the mice from each group were recorded daily (**Figure 2A** and **2C**) for 14 days. As depicted in the survival plot in **Figure 2B** and **2D**, the rates of survival declined with time in both the cyclodextrin-HM (35 mg/kg) and PVP-HM (30 mg/kg) treatment groups. No clear changes in body weight or physical condition were observed at 5, 10, and 15 mg/kg doses. However, significant decreases in body weight and increased signs of toxicity were observed in animals treated with both HM formulations at 20 or 25 mg/kg. Death was also observed at doses of both 30 and 35 mg/kg for both HM formulations. According to the above results, the maximum tolerated doses (MTD) for PVP-HM and cyclodextrin-HM were determined to be 20 and 30 mg/kg, respectively. Because the MTD of the cyclodextrin-HM formulation was the higher of the two, this formulation was selected for subsequent investigations.

### PK study of cyclodextrin-HM

**Figure 3:**
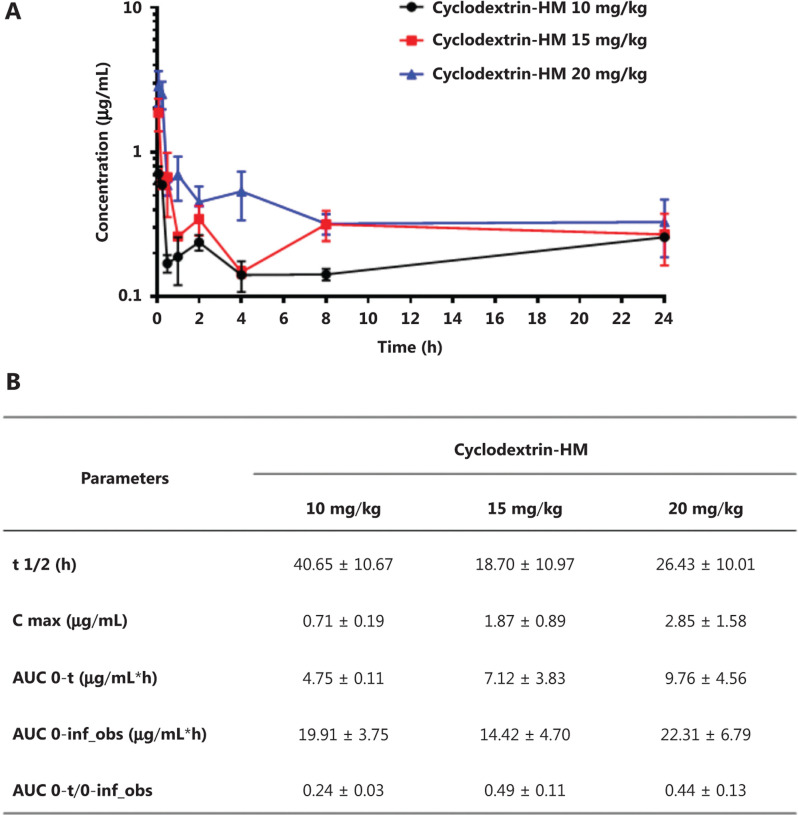
Mean plasma concentration/time profile after i.v. administration of the cyclodextrin-HM formula. (A) C57BL/6 mice were divided into 3 groups receiving different concentrations of cyclodextrin-HM (black, 10 mg/kg; red, 15 mg/kg; blue, 20 mg/kg). The concentrations of cyclodextrin-HM in the blood samples were measured at 0, 0.083, 0.25, 0.5, 1, 2, 4, 8, and 24 h after administration. (B) Pharmacokinetic parameters for assessing the plasma concentrations of the cyclodextrin-HM formula after i.v. injection. All data are presented as mean ± SD; *n* = 3.

To determine the PK parameters of HM, we administered mice 3 different doses of cyclodextrin-HM (10, 15, and 20 mg/kg) through i.v. injection *via* the tail vein. Blood samples were collected for measurement of HM in the plasma. LC/MS, and PK data were analyzed with non-compartmental analysis (**Figure 3A**). The half-life (t_1/2_) of HM was determined as 18.70 ± 10.97 to 40.65 ± 10.67 h, and the area under the curve (AUC_0-t_) values ranged from 4.75 ± 0.11 to 9.76 ± 4.56 μg/mL×h (**Figure 3B**). The PK of HM was dose-dependent (**Figure 3B**). Therefore, on the basis of PK and MTD data, 15 mg/kg was selected as the dose for subsequent studies.

### PK and dosing interval studies for co-treatment with cyclodextrin-HM and PTX

**Figure 4:**
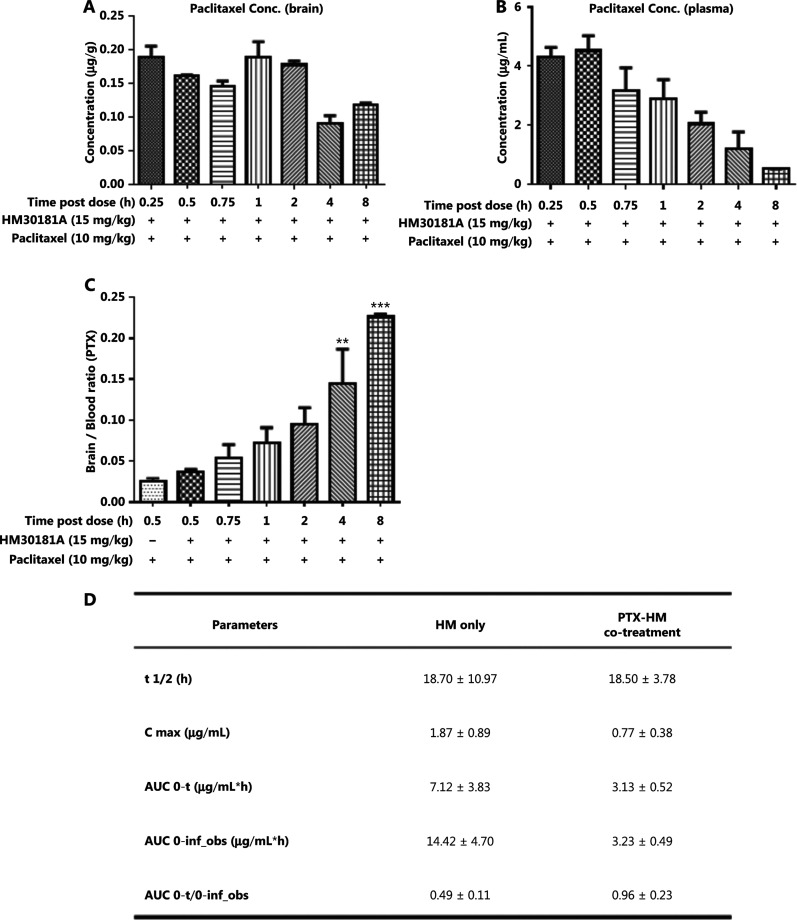
Optimum time interval for PTX injection after injection of cyclodextrin-HM. Mice were injected intravenously with cyclodextrin-HM (15 mg/kg) at 0.25, 0.5, 0.75, 1, 2, 4, and 8 h. All mice were treated with 10 mg/kg PTX 30 min after cyclodextrin-HM treatment at each time point. Plasma and brain samples were collected 0.5 h after i.v. administration of PTX. (A) Brain concentrations of PTX at the indicated time intervals. (B) Plasma concentrations of PTX after i.v. administration. All data are presented as mean ± SEM; *n* = 3. (C) Brain/blood ratio of PTX with the indicated treatments at the indicated time points. (D) Pharmacokinetic parameters of i.v. cyclodextrin-HM as a single agent (at 24 h) or in combination with PTX 10 mg/kg treatment (at 72 h).

To identify any potential drug-drug interactions when cyclodextrin-HM was co-administered with a chemotherapeutic drug, such as PTX, i.v. PTX at a dose of 10 mg/kg was administered to mice *via* the tail vein 30 min post-administration (i.v.) of cyclodextrin-HM (15 mg/kg). Plasma samples were collected at 72 h for the analysis of PK parameters in PTX-HM treatment (**Figure 4D**). The AUC 0-t/0-inf_obs value of 0.96 provided us with a rationale for optimizing the drug treatment time interval in *in vivo* experiments. As shown in **Figure 4D**, although the t_1/2_ value of cyclodextrin-HM with the addition of PTX at 18.7 h was comparable to the t_1/2_ value of cyclodextrin-HM treatment alone in plasma samples (18.5 h), a clear decrease was observed in the values of Cmax and AUC0-t in PTX-HM treatment, thus suggesting that the binding affinity of PTX (**Supplementary Figure S2**) toward plasma albumin may contribute to the decrease in these values. **Figure 4A** and **4B** show the plasma and brain concentrations of PTX among various dosing intervals, with PTX administered 0.25, 0.5, 0.75, 1, 2, 4, and 8 h after the administration of cyclodextrin-HM. Analysis of the collected brain and plasma samples revealed that HM facilitated higher levels of PTX when injected between 0.25 and 2 h post-administration of cyclodextrin-HM. Furthermore, as shown in **Figure 4C**, the brain/blood ratio for PTX increased in a time-dependent manner, thus suggesting a time-dependent increase in the bioavailability of PTX to the brain upon treatment with HM. To obtain the optimal concentration of PTX in blood and brain tissues, we selected 0.5 h as the interval for i.v. injection of PTX post-administration of cyclodextrin-HM.

### Brain penetration studies for co-treatment with cyclodextrin-HM and PTX

**Figure 5:**
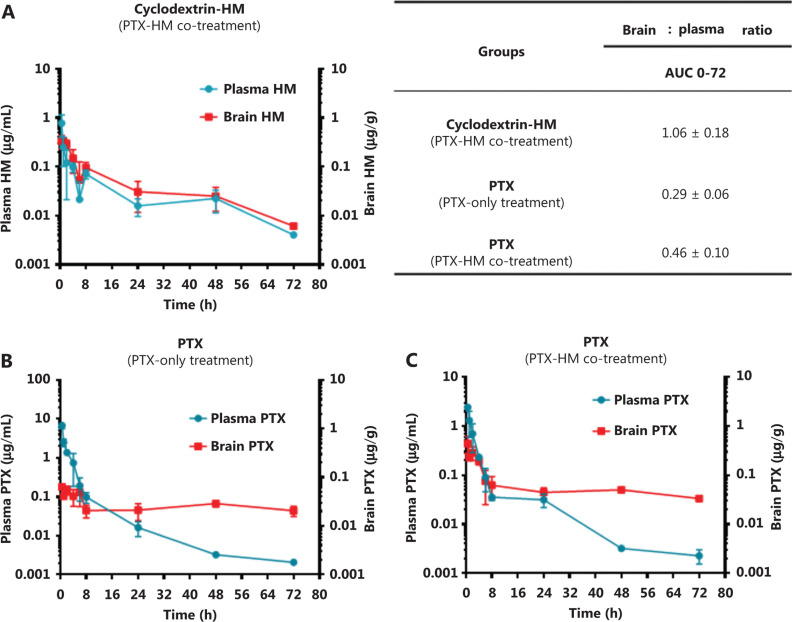
Effects of cyclodextrin-HM on the accumulation of PTX in the brain and plasma. Mice were intravenously administered cyclodextrin-HM (15 mg/kg), except for the control group. PTX (10 mg/kg) was injected into all groups *via* the tail vein 30 min post cyclodextrin-HM administration. The brain-to-plasma concentration ratios of compounds were measured. (A) Accumulation of cyclodextrin-HM in the brain and plasma. All data are presented as mean ± SD; *n* = 3. (B) Accumulation of PTX in the brain and plasma without administration of cyclodextrin-HM (control group). (C) Accumulation of PTX after co-treatment with PTX and cyclodextrin-HM. Plasma and brain samples were collected at 0.5, 1, 2, 4, 6, 8, 24, 48, and 72 h after intravenous administration.

The pharmacokinetic results in **Figure 3** indicated that the ratio of AUC 0-t/0-inf obs was less than 50% (ranging from 24% to 49%), thus revealing that HM was not completely metabolized within the set 24 h time point. Therefore, we examined time points beyond 24 h in the subsequent drug efficacy experiment, with monitoring time points at 48 and 72 h (**Figure 4D**). The AUC 0-t/0-inf_obs ratio at 72 h reached 96%, thus suggesting almost complete metabolization of HM. The ability of HM to increase PTX penetration into the brain was evident. The percentage penetration of a drug into the brain was expressed as brain AUC_0-72hr_ /plasma AUC_0-72hr_. The cyclodextrin-HM brain-to-plasma AUC ratio (AUC_brain_/AUC_plasma_) in mice injected i.v. with HM (15 mg/kg) between 0 and 72 h was determined to be 1.06 (0-last) and 1.08 (0-infinity), respectively (**Figure 5A**). Because the value of 0-last closely approximated the value of 0-infinity, most cyclodextrin-HM was metabolized in the brain and plasma within 72 h. Therefore, 0 to 72 h was selected as the optimal time duration for PK studies of PTX in the blood and the brain. At 0.5 h post-administration of the control solvent or cyclodextrin-HM, PTX at a dose of 10 mg/kg was injected i.v. into the mice, and plasma and brain samples were collected between 0 and 72 h after the administration of PTX. Whereas the PTX brain-to-plasma AUC ratio (AUC_brain_/AUC_plasma_) was 0.29 in the control group (**Figure 5B**), the ratio was higher (0.46) for PTX with administration of cyclodextrin-HM (**Figure 5C**). The shift in the brain-to-plasma ratio in the presence of HM thus increases the likelihood of efficacious concentrations of PTX entering the brain.

### Co-treatment with cyclodextrin-HM and PTX in an orthotopic brain tumor model

**Figure 6:**
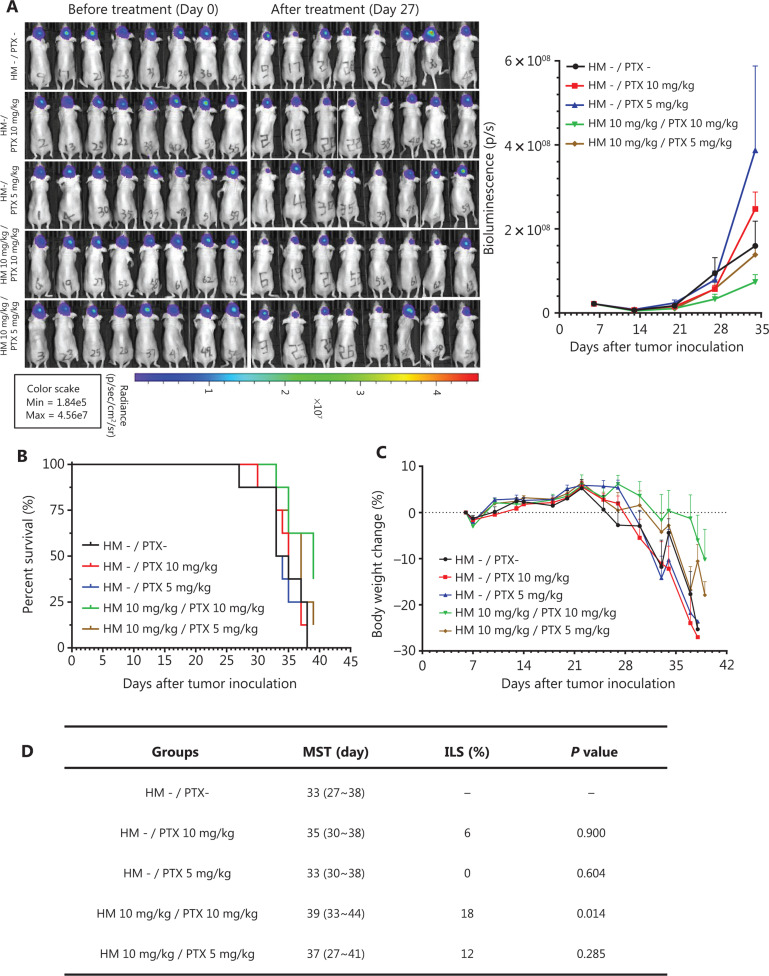
Cyclodextrin-HM promoted PTX-induced inhibition of orthotopic brain tumor growth *in vivo*. Mice were divided into 5 groups: co-treatment (indicated as brown and green); PTX treatment without cyclodextrin-HM administration (indicated as blue and red); and untreated control (indicated as black). (A) Bioluminescence image of mice before and after treatment (left panel), and analysis of the average intensity of the bioluminescence signal during the course of treatment (right panel). Data are presented as mean ± SEM. (B and C) Survival rates and changes in body weights of mice during the experimental period. (D) Survival data for cyclodextrin-HM plus PTX treatment.

The therapeutic efficacy of the co-administration of cyclodextrin-HM and PTX was validated with an orthotopic glioma mouse model. After i.c. inoculation with U-87 MG-Luc2 glioma cells of human origin, mice were randomly divided into 5 groups: i) vehicle control (with vehicles for PTX and HM); ii) and ii) PTX treatment (pre-administration with vehicles of HM); and iv) and v) PTX and cyclodextrin-HM co-treatment. Two concentrations (5 and 10 mg/kg) of PTX were administered, and 10 mg/kg cyclodextrin-HM was used. The intensity of the bioluminescence signal quantifying the tumor size was analyzed (**Figure 6A**). Although discrepancies were observed between treatment with vehicle and PTX alone, possibly because of varying susceptibility to tumor development among individual mice, a comparison of the vehicle control and PTX treatment groups revealed that the bioluminescence signal detected in mice co-administered cyclodextrin-HM and PTX (10 mg/kg) decreased significantly in a dose-dependent manner, thus indicating that cyclodextrin-HM enhanced the anti-tumor effect of PTX. However, the intensity of bioluminescence in the co-treatment group receiving 5 mg/kg of PTX was similar to that in the vehicle control and PTX treatment groups. Consistently with this finding, the lifespan of the mice after co-treatment with 10 mg/kg (not with 5 mg/kg) of PTX was significantly longer than that in mice in the PTX treatment group, as shown in **Figure 6B**. **Figure 6D** shows that the MST of the co-treated mice was 39 days (ILS, 18%; *P* = 0.014) and 37 days (ILS, 12%; *P* = 0.285) for treatment with cyclodextrin-HM with 10 and 5 mg/kg PTX, respectively. For mice in the PTX treatment group receiving 10 and 5 mg/kg PTX, the MST was 35 (ILS, 6%; *P* = 0.900) and 33 days (ILS, 0%; *P* = 0.604), respectively. In addition, the survival time of the co-treatment group receiving 10 mg/kg PTX increased and was also significantly different from that in the group receiving vehicle control (*P* = 0.014), as well as the PTX treatment groups receiving the same dosage of PTX (*P* = 0.018). Therefore, i.v. administration of cyclodextrin-HM (10 mg/kg), with subsequent injection of PTX (10 mg/kg), is an effective formulation for treating the progression of brain tumors in the xenograft model of glioblastoma.

## Discussion

Drug resistance arising from the persistent administration of PTX is a critical factor affecting the successful treatment of different types of cancers. One cause of this insensitivity to therapeutic agents is the activation of cellular efflux mechanisms, which lead to inefficient drug delivery. Because P-gp is a major cellular pump that removes foreign substances and delivers them into the blood, body fluids, and the intestinal lumen^19^, the focus of the present study was to explore a novel injectable formulation to facilitate the delivery of PTX through co-administration of a P-gp inhibitor for the treatment of glioma. Many small molecules able to modulate P-gp activity have been discovered, e.g., cyclosporine and verapamil. However, cyclosporine, a prescribed immunosuppressant approved by the Food and Drug Administration, has low affinity toward P-gp^20^. The effective dose of cyclosporine, which antagonizes the transport function of P-gp, is high: at or above 1.3 μM^21–23^. A clinical trial of verapamil has encountered the same problem^24^. The results from previous studies illustrate the practical difficulties of using these compounds, owing to the adverse effects and toxicity associated with high-dose administration.

In the present study, the use of HM was investigated for inhibiting P-gp activity. HM is of particular interest because it has the highest potency in inhibiting the transepithelial transport of PTX among other P-gp inhibitors, such as cyclosporin A, elacridar, and tariquidar, in Madin-Darby canine kidney cell monolayers transfected with P-gp^8^, which is a cellular model for identifying human P-gp substrates and inhibitors^25–27^. In addition, mice with intra-cerebrally implanted tumor cells show increased apoptosis of tumor cells in brain tumors after co-oral administration of HM and PTX. A recent phase I clinical study investigating the use of an oral drug containing PTX and HM for the treatment of advanced solid tumors, such as cancers of the breast, pancreas, and gallbladder, has suggested the relative safety of HM PTX co-treatment in clinical practice ^28^.

Because the bioavailability of HM is low after i.v. administration, owing to its insolubility in water and organic solvents, HM was formulated with different excipients (i.e., PVP and HP-β-cyclodextrin) by using semi-solid dispersion and particle size reduction. HM dissolved in either PVP-HM or cyclodextrin-HM showed no significant differences in inhibiting the P-gp activity of the human A549 lung carcinoma cell line (**Figure 1**). However, cyclodextrin-HM demonstrated higher physical stability at room temperature and tolerability when applied as multiple injections in C57BL/6 mice (**Figure 2**). Of note, PVP and cyclodextrin may also enhance the inhibitory effect of HM *via* the modulation of P-gp activity, similarly to other polymers, such as F127^29,30^. The absorption of genipin by the intestine has been found to be facilitated by a cyclodextrin-induced decrease in P-gp ATPase activity^31^. Cyclodextrin can also enhance the accumulation of nanoparticles of PTX in multidrug-resistant breast cancer cells overexpressing P-gp^32^. The P-gp inhibitory effect of PVP has also been documented^33^. In this study, cyclodextrin-HM, instead of PVP-HM, was chosen for subsequent PK and pharmacological investigations in multiple mouse models, because the high viscosity of PVP-HM affected the i.v. injection procedure. In fact, cyclodextrin is a suitable excipient because of its ability to form complexes with drugs of interest, particularly inclusion particles, which protect the drug molecule in the hydrophobic cavity^34^. Such characteristics of cyclodextrins sustain drug pharmacological activity by enabling solubility in aqueous solution without sacrificing lipophilicity for passive diffusion across biological membranes^34^. In addition, cyclodextrin-HM has an effective concentration in the nanomolar range (50–500 nM) *in vitro*, which is significantly lower than those of other well-known P-gp inhibitors, such as cyclosporine and verapamil, whose therapeutically effective concentrations are in the micromolar range. Therefore, the data from the present study suggest a potential decrease in adverse effects during the clinical application of cyclodextrin-HM.

The blockage of the delivery of most therapeutics into the brain by the blood-brain barrier remains one of the major barriers to the exploitation of pharmaceutical intervention targeting brain tumors^35^. Over the past several years, transport of anti-cancer drugs to penetrate the blood-brain barrier has been successfully achieved, including the delivery of nanoparticle-based drugs through strategies involving different types of transcytosis, noninvasive intranasal administration, and temporal opening of the blood-brain barrier through physical and chemical means^36,37^ to effectively elevate the cerebral concentration of the administered drugs. However, poor selectivity of the nanodrugs after they reach the brain upon delivery *via* these methods leads to major concerns regarding drug promiscuity^38^. In our findings, the PK and pharmacological analyses of the present study revealed that i.v. injection of cyclodextrin-HM improved the delivery of PTX across the blood-brain barrier (**Figure 5**) and specifically decreased the tumor burden (**Figure 6**). In addition, these results suggest that the improved therapeutic effect of PTX is partially due to the inhibited activity of P-gp expressed on the capillary endothelial cells at the blood-brain barrier. The reduced tumor sizes may also be associated with increased accumulation of PTX in glioma cells, owing to the inhibition of cancer-expressed P-gp, although this possibility that requires further verification. On the basis of the current findings, cyclodextrin-HM appears to be a favorable P-gp inhibitor formulation for combined use with anti-cancer agents, such as PTX. To date, successful small molecule P-gp inhibitors or modulators are scarce. The wide distribution of P-gp and its protective role in normal tissues can render P-gp inhibitors toxic in clinical use^20^. The existence of multiple binding sites in P-gp also hinders the search for potent compounds for inhibiting the activity of P-gp^20^.

**Figure 7:**
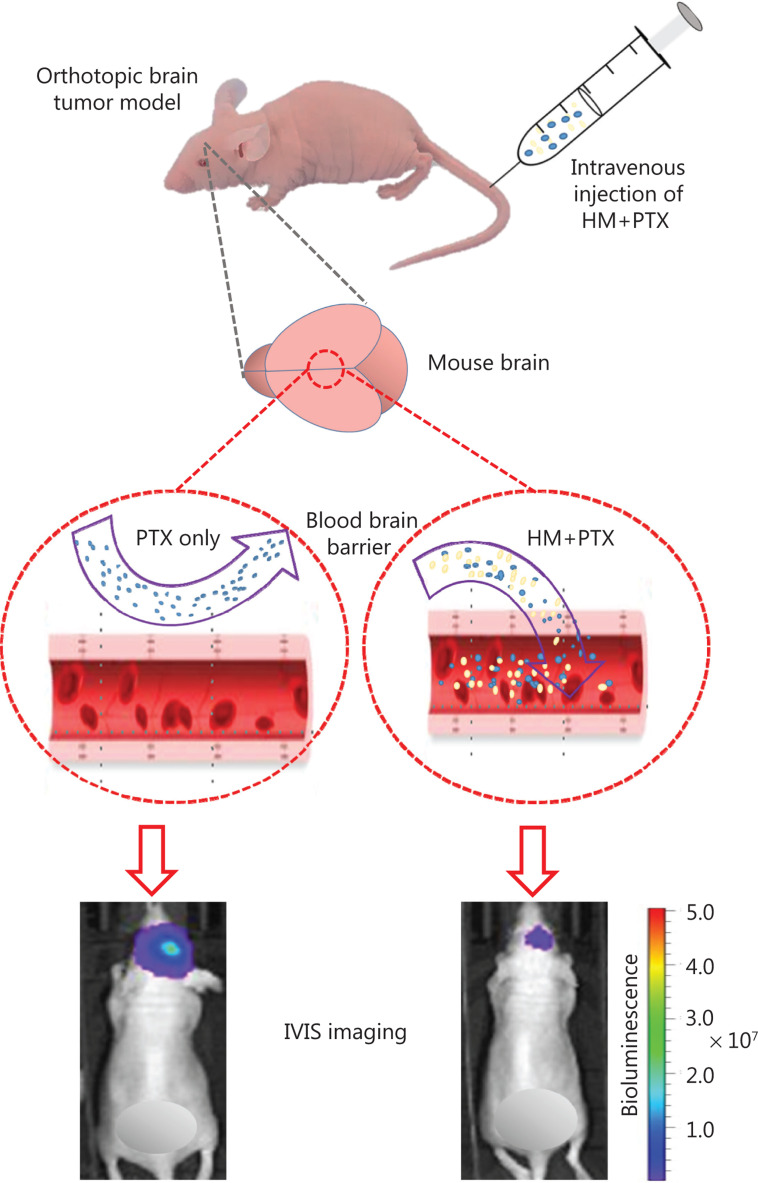
Optimized intravenous formulation of solubilized HM30181A (HM) to inhibit P-glycoprotein (P-gp) in the brain for effective cerebral delivery of paclitaxel (PTX) for the treatment of malignant glioma. In the mouse orthotopic brain tumor model, co-administration of cyclodextrin-HM with PTX showed potent anti-tumor effects, thus implicating a potential clinical pharmacological strategy for treating malignant glioma.

Here, a cyclodextrin-based HM formulation was developed that shows enhanced solubility and substantial therapeutic efficacy in a murine model of orthotopic glioma. The formulation preserved the efficacy of HM in inhibiting P-gp, thus promoting the penetration of PTX into the brain (**Figure 7**). The findings from the present study also provide useful information supporting the exploration and improvement of pharmacological delivery methods for different chemotherapeutics by manipulating the P-gp efflux process *via* combined treatment with HM as a therapy for other types of cancer.

## Conclusions

In the current study, an optimized intravenous formulation of solubilized HM that is able to inhibit P-gp in the brain for effective cerebral delivery of PTX was developed for the treatment of malignant glioma. In a mouse orthotopic brain tumor model, co-administration of cyclodextrin-HM with PTX was confirmed to have potent anti-tumor effects *via* increasing the oral bioavailability and efficacy of PTX. Therefore, this formulation could be further developed for treating malignant glioma in humans.

## References

[1] CBTRUS Statistical Report (2010). Primary brain and central nervous system tumors diagnosed in the United States in 2004–2006.

[2] Fellner S, Bauer B, Miller DS, Schaffrik M, Fankhanel M, Spruss T (2002). Transport of paclitaxel (Taxol) across the blood-brain barrier in vitro and in vivo. J Clin Invest.

[3] Jibodh RA, Lagas JS, Nuijen B, Beijnen JH, Schellens JH (2013). Taxanes: old drugs, new oral formulations. Eur J Pharmacol.

[4] Hendrikx JJ, Beijnen JH, Schinkel AH (2014). P-gp and taxanes. Oncoscience.

[5] Zhen Z, Yang K, Ye L, You Z, Chen R, Liu Y (2017). Suberoylanilide hydroxamic acid sensitizes neuroblastoma to paclitaxel by inhibiting thioredoxin-related protein 14-mediated autophagy. Cancer Sci.

[6] Joo KM, Park K, Kong DS, Song SY, Kim MH, Lee GS (2008). Oral paclitaxel chemotherapy for brain tumors: ideal combination treatment of paclitaxel and P-glycoprotein inhibitor. Oncol Rep.

[7] Fox E, Bates SE (2007). Tariquidar (XR9576): a P-glycoprotein drug efflux pump inhibitor. Expert Rev Anticancer Ther.

[8] Kwak JO, Lee SH, Lee GS, Kim MS, Ahn YG, Lee JH (2010). Selective inhibition of MDR1 (ABCB1) by HM30181 increases oral bioavailability and therapeutic efficacy of paclitaxel. Eur J Pharmacol.

[9] Bühler V (2005). Polyvinylpyrrolidone excipients for pharmaceuticals: povidone, crospovidone and copovidone.

[10] Uekama K (2004). Design and evaluation of cyclodextrin-based drug formulation. Chem Pharm Bull (Tokyo).

[11] Parikh DM (2016). Handbook of pharmaceutical granulation technology.

[12] McDonald TA, Zepeda ML, Tomlinson MJ, Bee WH, Ivens IA (2010). Subcutaneous administration of biotherapeutics: current experience in animal models. Curr Opin Mol Ther.

[13] Kulkarni AD, Patel HM, Surana SJ, Belgamwar VS, Pardeshi CV (2016). Brain-blood ratio: implications in brain drug delivery. Expert Opin Drug Deliv.

[14] Hsiao P, Sasongko L, Link JM, Mankoff DA, Muzi M, Collier AC (2006). Verapamil p-glycoprotein transport across the rat blood-brain barrier: cyclosporine, a concentration inhibition analysis, and comparison with human data. J Pharmacol Exp Ther.

[15] Zhang Y, Huo M, Zhou J, Xie S (2010). Pksolver: an add-in program for pharmacokinetic and pharmacodynamic data analysis in microsoft excel. Comput Methods Programs Biomed.

[16] Bailer AJ (1988). Testing for the equality of area under the curves when using destructive measurement techniques. J Pharmacokinet Biopharm.

[17] Vaidyanathan A, Sawers L, Gannon AL, Chakravarty P, Scott AL, Bray SE (2016). ABCB1 (MDR1) induction defines a common resistance mechanism in paclitaxel- and olaparib-resistant ovarian cancer cells. Br J Cancer.

[18] Jouan E, Le Vee M, Mayati A, Denizot C, Parmentier Y, Fardel O (2016). Evaluation of P-glycoprotein inhibitory potential using a rhodamine 123 accumulation assay. Pharmaceutics.

[19] Wessler JD, Grip LT, Mendell J, Giugliano RP (2013). The P-glycoprotein transport system and cardiovascular drugs. J Am Coll Cardiol.

[20] Waghray D, Zhang Q (2018). Inhibit or evade multidrug resistance P-glycoprotein in cancer treatment. J Med Chem.

[21] Coley HM, Twentyman PR, Workman P (1989). Improved cellular accumulation is characteristic of anthracyclines which retain high activity in multidrug resistant cell lines, alone or in combination with verapamil or cyclosporin A. Biochem Pharmacol.

[22] Nooter K, Sonneveld P, Oostrum R, Herweijer H, Hagenbeek T, Valerio D (1990). Overexpression of the mdr1 gene in blast cells from patients with acute myelocytic leukemia is associated with decreased anthracycline accumulation that can be restored by cyclosporin-A. Int J Cancer.

[23] Naito M, Tsuge H, Kuroko C, Koyama T, Tomida A, Tatsuta T (1993). Enhancement of cellular accumulation of cyclosporine by anti-P-glycoprotein monoclonal antibody MRK-16 and synergistic modulation of multidrug resistance. J Natl Cancer Inst.

[24] Hollt V, Kouba M, Dietel M, Vogt G (1992). Stereoisomers of calcium antagonists which differ markedly in their potencies as calcium blockers are equally effective in modulating drug transport by P-glycoprotein. Biochem Pharmacol.

[25] Yamazaki M, Neway WE, Ohe T, Chen I, Rowe JF, Hochman JH (2001). In vitro substrate identification studies for p-glycoprotein-mediated transport: species difference and predictability of in vivo results. J Pharmacol Exp Ther.

[26] Polli JW, Wring SA, Humphreys JE, Huang L, Morgan JB, Webster LO (2001). Rational use of in vitro P-glycoprotein assays in drug discovery. J Pharmacol Exp Ther.

[27] Pastan I, Gottesman MM, Ueda K, Lovelace E, Rutherford AV, Willingham MC (1988). A retrovirus carrying an MDR1 cDNA confers multidrug resistance and polarized expression of P-glycoprotein in MDCK cells. Proc Natl Acad Sci U S A.

[28] Lee HJ, Heo DS, Cho JY, Han SW, Chang HJ, Yi HG (2014). A phase I study of oral paclitaxel with a novel P-glycoprotein inhibitor, HM30181a, in patients with advanced solid cancer. Cancer Res Treat.

[29] Guan Y, Huang J, Zuo L, Xu J, Si L, Qiu J (2011). Effect of pluronic P123 and F127 block copolymer on P-glycoprotein transport and CYP3A metabolism. Arch Pharm Res.

[30] Wei Z, Yuan S, Hao J, Fang X (2013). Mechanism of inhibition of P-glycoprotein mediated efflux by Pluronic P123/F127 block copolymers: relationship between copolymer concentration and inhibitory activity. Eur J Pharm Biopharm.

[31] Zhang Y, Meng FC, Cui YL, Song YF (2011). Enhancing effect of hydroxypropyl-beta-cyclodextrin on the intestinal absorption process of genipin. J Agric Food Chem.

[32] Baek JS, Cho CW (2013). 2-hydroxypropyl-beta-cyclodextrin-modified SLN of paclitaxel for overcoming p-glycoprotein function in multidrug-resistant breast cancer cells. J Pharm Pharmacol.

[33] Rehman S, Nabi B, Fazil M, Khan S, Bari NK, Singh R (2017). Role of P-glycoprotein inhibitors in the bioavailability enhancement of solid dispersion of darunavir. Biomed Res Int.

[34] Gidwani B, Vyas A (2015). A comprehensive review on cyclodextrin-based carriers for delivery of chemotherapeutic cytotoxic anticancer drugs. Biomed Res Int.

[35] Pardridge WM (2002). Drug and gene targeting to the brain with molecular Trojan horses. Nat Rev Drug Discov.

[36] Wong HL, Wu XY, Bendayan R (2012). Nanotechnological advances for the delivery of cns therapeutics. Adv Drug Deliv Rev.

[37] Li D, Kerns EH (2015). Blood-brain barrier in drug discovery: optimizing brain exposure of cns drugs and minimizing brain side effects.

[38] Gao H (2017). Perspectives on dual targeting delivery systems for brain tumors. J Neuroimmune Pharmacol.

