# Supplementary material for: HM30181A, a potent P-glycoprotein inhibitor, potentiates the absorption and in vivo antitumor efficacy of paclitaxel in an orthotopic brain tumor model
Source: Cancer Biol Med. 2020 Dec 15;17(4):986–1001. doi: 10.20892/j.issn.2095-3941.2020.0128 (PMC7721091; doi:10.20892/j.issn.2095-3941.2020.0128)
Supplement: Supplementary file 1 [file cbm-17-986-s001.pdf]

Supplementary materials

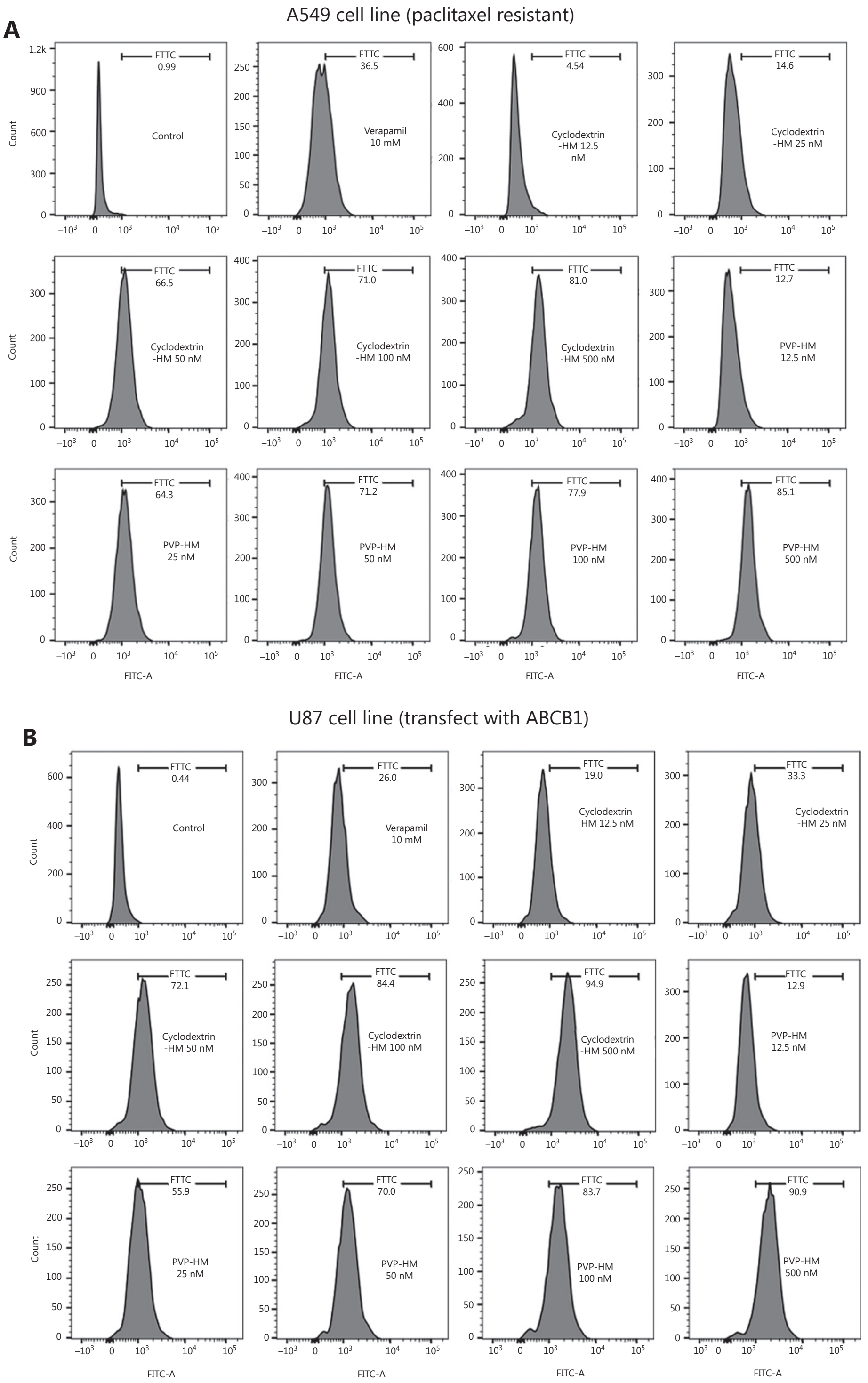

Figure S1 (A) Mean fluorescence intensity of Rho123 in PTX-resistant A549 cancer cells detected by flow cytometry. (B) Mean fluorescence intensity of Rho123 in ABCB1 overexpressing U-87 MG cells detected by flow cytometry.

A

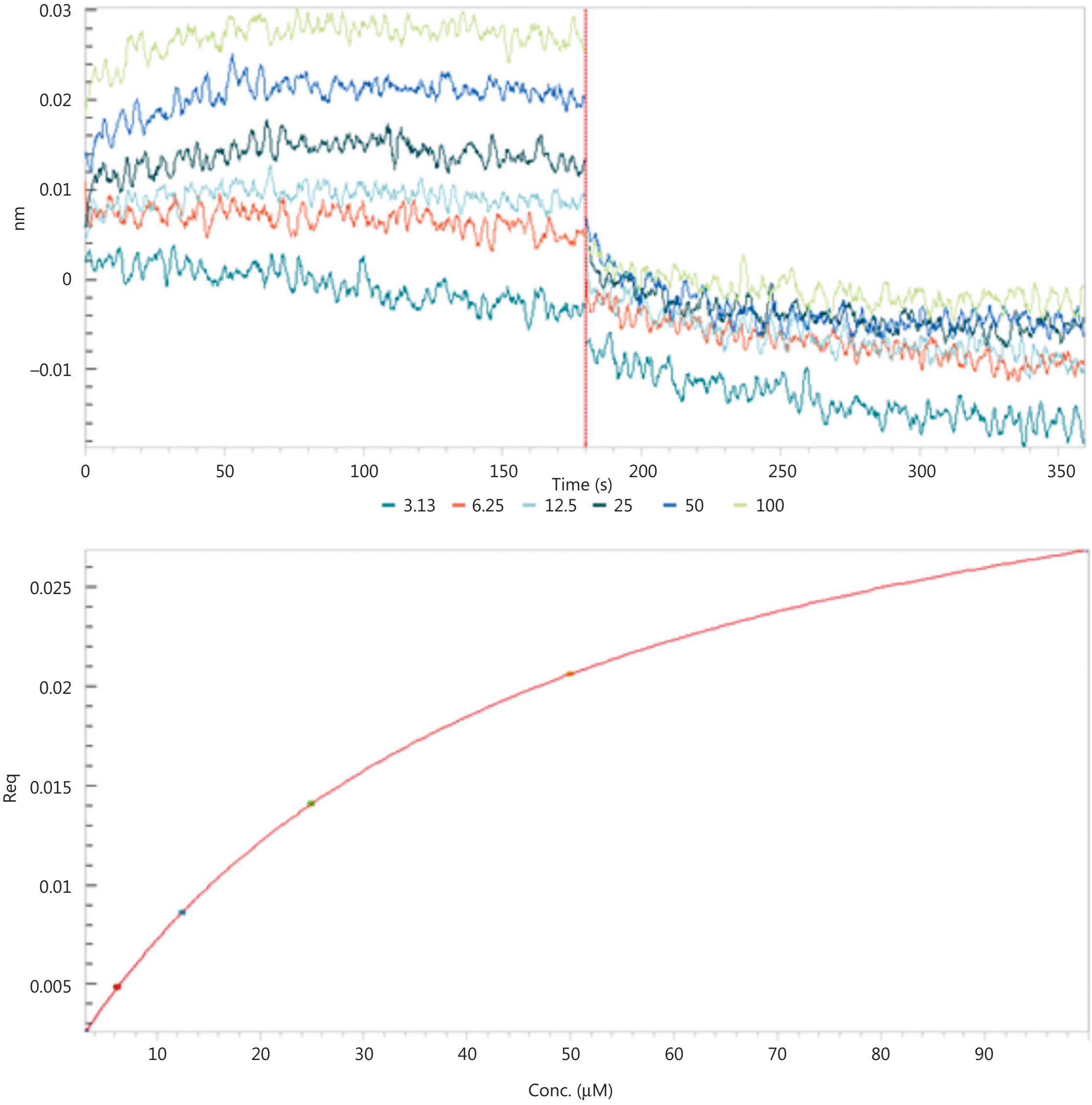

KD (M)	kon(1/Ms)	kdis(1/s)	R^2
4.33E-05	2.65E+04	1.15E+00	0.7875

Figure S2 Bio-layer interferometry analysis. The binding affinity of PTX and cyclodextrin-HM toward the plasma albumin protein was assessed with bio-layer interferometry (BLI) technology. In brief, whereas (A) the association constant (K_D) of PTX toward albumin was 4.33×10^{-5} (43 µM), and (B) the association K_D of cyclodextrin-HM toward albumin was similar to the value of 4.64×10^{-5} (46.4 µM).

B

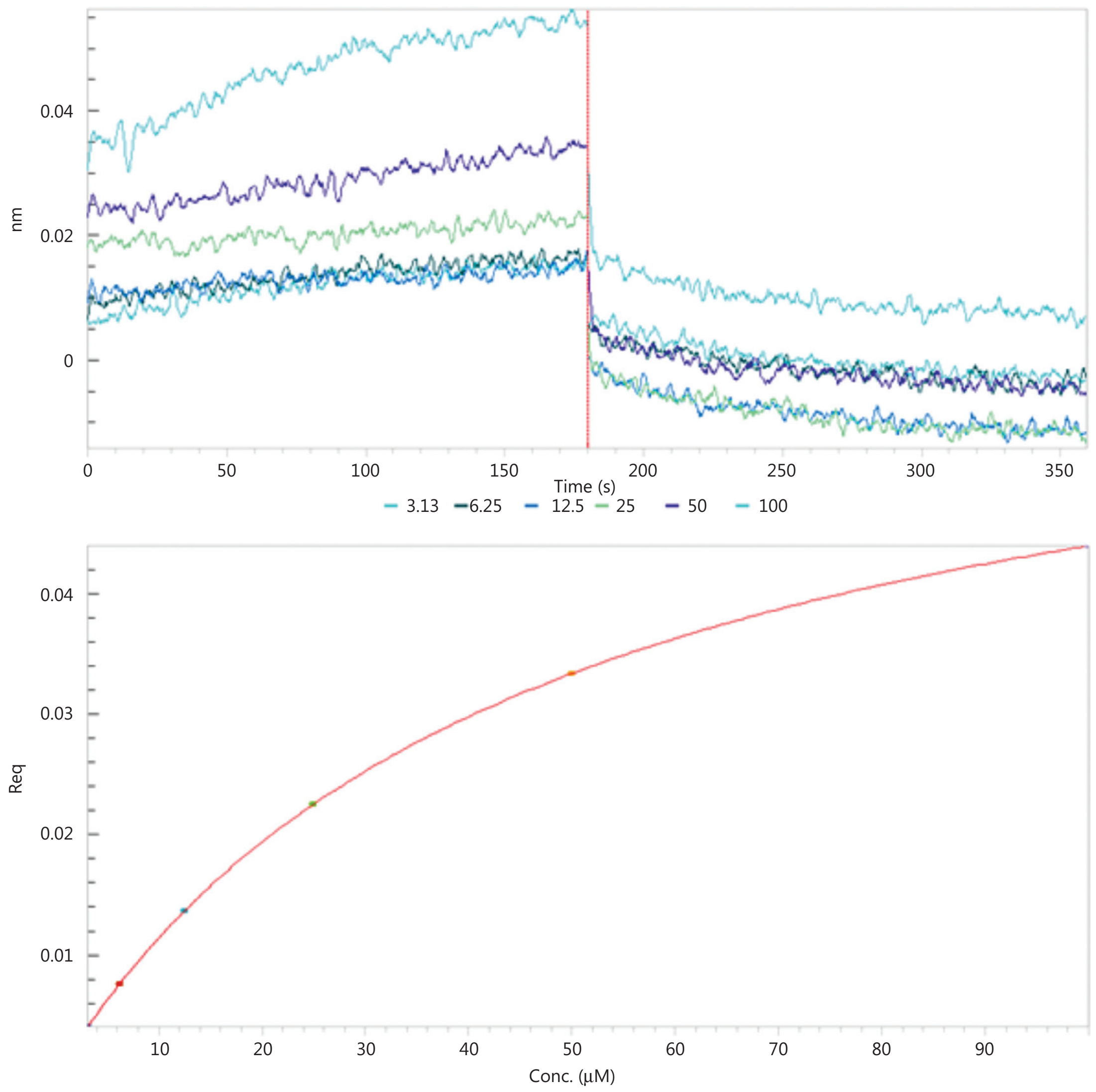

KD (M)	kon(1/Ms)	kdis(1/s)	R^2
4.64E-05	1.07E+04	4.98E-01	0.8361

Figure S2 (continue)

Table S1 Calibration equations, correlation coefficients (R^2), linear ranges LOD, LOQ, and recovery of HM-Brain and HM-Plasma

Compound	Calibration equations	R^2	Linear Range (μg/L)	LOD (μg/L)	LOQ (μg/L)	Low (n = 6)		Medium (n = 6)		High (n = 6)	
						Recovery (%)	RSD (%)	Recovery (%)	RSD (%)	Recovery (%)	RSD (%)
HM-Brain	Y = 22007X + 6160.3	R^2 = 0.996	0.00036–0.11472	0.0115	0.115	93.6 ± 12.5	13.6	106 ± 15.6	14.7	103 ± 13.3	12.9
HM-Plasma	Y = 75672X–7561.6	R^2 = 0.996	0.0001–0.00736	0.00115	0.0575	113 ± 15.3	13.5	113 ± 18.5	16.4	109 ± 11.5	10.6

All data are shown as mean ± SD at each spiking level. At each level, 3 replicates and 2 measurements were used (n = 6). The recovery rate was computed as the average recovery of 3 spiking levels.
